# Prevalence of *Mycobacterium kansasii* in clinical and environmental isolates, a systematic review and meta-analysis

**DOI:** 10.3389/fmicb.2024.1321273

**Published:** 2024-02-19

**Authors:** Negar Narimisa, Narjess Bostanghadiri, Forough Goodarzi, Shabnam Razavi, Faramarz Masjedian Jazi

**Affiliations:** ^1^Department of Microbiology, School of Medicine, Iran University of Medical Sciences, Tehran, Iran; ^2^Department of Bacteriology, Faculty of Medical Sciences, Tarbiat Modares University, Tehran, Iran

**Keywords:** *Mycobacterium kansasii*, meta-analysis, CMA, prevalence, NTM

## Abstract

**Background:**

*Mycobacterium kansasii* infection is one of the most common causes of non-tuberculosis mycobacterial (NTM) disease worldwide. However, accurate information on the global prevalence of this bacterium is lacking. Therefore, this study was conducted to investigate the prevalence of *M. kansasii* in clinical and environmental isolates.

**Methods:**

Databases, including PubMed, Scopus, and the Web of Science, were utilized to gather articles on the prevalence of *M. kansasii* in clinical and environmental isolates. The collected data were analyzed using Comprehensive Meta-Analysis software.

**Results:**

A total of 118 and 16 studies met the inclusion criteria and were used to analyze the prevalence of *M. kansasii* in clinical and environmental isolates, respectively. The prevalence of *M. kansasii* in NTM and environmental isolates were 9.4 and 5.8%, respectively. Subsequent analysis showed an increasing prevalence of *M. kansasii* over the years. Additionally, the results indicated a significant difference in the prevalence of this bacteria among different regions.

**Conclusion:**

The relatively high prevalence of *M. kansasii* among NTM isolates suggests the need for further implementation of infection control strategies. It is also important to establish appropriate diagnostic criteria and management guidelines for screening this microorganism in environmental samples in order to prevent its spread, given its high prevalence in environmental isolates.

## Introduction

The genus *Mycobacterium* comprises over 200 species, divided into the *Mycobacterium tuberculosis* (MTB) complex and non-tuberculosis mycobacteria (NTM) (Karami-Zarandi et al., 2019). NTM is a diverse group of opportunistic bacteria that are commonly found in water, soil, and dust. While tuberculosis (TB) is the most prevalent mycobacterial infection in developing countries, the incidence of NTM diseases is rising globally, surpassing tuberculosis in developed nations (Johansen et al., 2020; Pavlik et al., 2022).

Initially, NTMs were considered contaminants rather than pathogens due to their presence in environmental sources (Koh, 2017). However, the incidence of NTM diseases has increased, and the exact cause of this rise remains poorly understood. Factors such as an aging population, reduced immune function, and environmental exposure to mycobacteria have been suggested as possible explanations (Cowman et al., 2019).

*Mycobacterium kansasii* (*M. kansasii*) is a slow-growing NTM that causes pulmonary and extra-pulmonary infections, in immunocompromised and immunocompetent individuals (Khosravi et al., 2020). The disease caused by *M. kansasii* closely resembles pulmonary tuberculosis in terms of pathogenesis, clinical features, and treatment response, differing significantly from infections caused by other NTM, particularly the *M. avium* complex (Woods and Washington, 1987).

Traditionally, *M. kansasii* has been recognized as an NTM pathogen causing lung disease rather than a contaminant. The isolation of *M. kansasii* from sputum under appropriate conditions may be sufficient evidence to indicate disease and to initiate treatment (Matveychuk et al., 2012; Daley et al., 2020).

Global reports have identified *M. kansasii* as the sixth most commonly isolated NTM from clinical samples. Additionally, it has been reported as the leading cause of pulmonary NTM disease in sub-Saharan Africa and the third most prevalent NTM causing lung disease in Taiwan (Huang et al., 2017; Okoi et al., 2017).

There is a widely held belief that *M. kansasii* can be acquired from the environment and is present in various natural ecosystems, including water, soil, and dust. Numerous studies have documented the recovery of this organism from municipal water distribution systems, with isolates found in the same communities where *M. kansasii* disease patients have been identified (McSwiggan and Collins, 1974; Steadham, 1980). The epidemiology of *M. kansasii* primarily affects urban areas, particularly high-density, low-income communities in highly industrialized regions (Kwenda et al., 2015).

Considering the clinical importance of *M. kansasii* and the lack of a meta-analysis study examining the prevalence of *M. kansasii* in clinical and environmental samples, the aim of this study is to investigate its prevalence in both clinical and environmental samples. The information obtained from this study can contribute to the effective management of this bacterium.

## Materials and methods

### Search strategy

We conducted a search of journal articles in three databases (PubMed, Scopus, and Web of Science) until February 2023. All of these databases were searched using the following search strategy: “*Mycobacterium kansasii*” OR “*M. kansasii*.”

### Eligibility criteria

All studies that provided the precise number of *M. kansasii* isolates - either as total isolates or as part of NTM isolates in clinical samples, as well as studies that reported the bacterial count in environmental samples, were included in this study.

All identified articles were collated using Endnote X20 Citation Manager Software, and duplicate articles were removed prior to review. The citations were then uploaded to Rayyan, a citation classification application (Ouzzani et al., 2016). Two independent reviewers screened the titles and abstracts, and removed irrelevant articles. Full texts of potentially relevant articles were independently collected and reviewed by two authors. If there was a disagreement about the inclusion of an article after screening, a third author determined its eligibility for full review.

Review articles, case report studies, short communications, conference papers, letters, book chapters, articles that did not mention the exact number of isolates, and articles written in languages other than English were excluded.

### Data extraction

**Figure 1:**
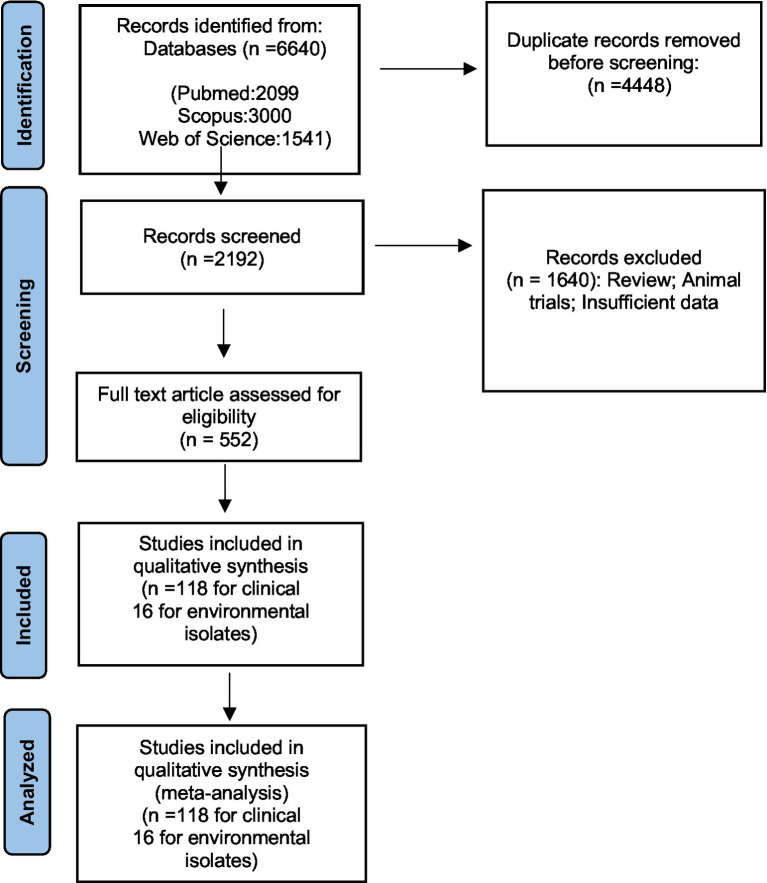
The study prisma flow diagram.

Two authors independently extracted all data from eligible articles. Any disagreements in data points were resolved through consensus and discussion. From each article, we collected information on the first author, publication year, sampling time, study country, continent, and sample size (total number of samples and number of M. *kansasi* in clinical and environmental samples). This study selection process was presented in a Preferred Reporting Item for Systematic Reviews and Meta-Analyses (PRISMA) flowchart (Figure 1).

### Study quality assessment

The quality of included studies was evaluated using the Joanna Briggs Institute (JBI) Critical Appraisal Checklist (Munn et al., 2020). This checklist consists of nine questions that assess the quality of studies, focusing on appropriate sampling techniques, research objectives, and adequate data analysis. Each item is rated as “yes,” “no,” or “unclear.” A score of 1 point is given for each “yes” answer, and a score of 0 points is given for each “no” or “unclear” answer. Finally, the mean score of each paper was independently evaluated by two reviewers, and any disagreements were resolved through consensus between the two reviewers or by consulting a third author, if needed.

### Data analysis

Data analysis on the prevalence of *M. kansasi* in clinics and the environment was conducted using Comprehensive Meta-analysis (CMA) software. The analysis included prevalence data for *M. kansasi* in NTM, clinical isolates, water isolates, and soil isolates.

Subgroup analyses were performed based on sampling period, country, continent, and method of detection for prevalence of *M. kansasi* in NTM clinical isolates. For water samples, subgroup analyses were conducted based on country, continent, and place of collection.

A random-effects model was utilized to estimate the pooled prevalence of *M. kansasi* in clinical and environmental samples with a 95% confidence interval. The heterogeneity among the studies in the meta-analysis was assessed using the I^2^ statistic. An I^2^ value of ≤25% indicates low homogeneity, 25% < I^2^ < 75% indicates moderate heterogeneity, and I^2^ > 75% indicates high heterogeneity.

Sensitivity analysis was performed to investigate the impact of individual studies on the prevalence of *M. kansasi* in NTM and environmental isolates. Funnel plots and Begg’s test were employed to assess the presence of publication bias. Results were considered to have publication bias if the *p*-value was <0.05.

## Results

### Search results

A total of 6,640 publications were identified. After removing duplicates using Endnote software, 2,192 articles were screened. Following the screening process, 1,640 studies were excluded, leaving 552 articles for full-text validation. After a thorough review, 118 published studies were used to analyze the prevalence of *M. kansasi* in clinical isolates (Wright et al., 1985; Debrunner et al., 1992; Rastogi and Goh, 1992; Shafer and Sierra, 1992; Parenti et al., 1995; Gamboa et al., 1997; Benjamin et al., 1998; Alcaide et al., 1999; Attorri et al., 2000; Ruiz et al., 2001; Mijs et al., 2002; Scarparo et al., 2002; Alcaide et al., 2003; Kontos et al., 2003; Rodriguez Díaz et al., 2003; Tu et al., 2003; Martin-Casabona et al., 2004; Matos et al., 2004; Morita et al., 2005; Pierre-Audigier et al., 2005; Prammananan et al., 2005; Dailloux et al., 2006; Franco-Álvarez de Luna et al., 2006; Hillemann et al., 2006; Lai et al., 2006; Andréjak et al., 2007; Liao et al., 2007; Bodle et al., 2008; Pedro et al., 2008; Ryoo et al., 2008; al-Mahruqi et al., 2009; Shen et al., 2009; Sorlozano et al., 2009; Amorim et al., 2010; Bicmen et al., 2010; Moore et al., 2010; Shenai et al., 2010; al Houqani et al., 2011; Ani et al., 2011; Bicmen et al., 2011; Chae et al., 2011; del Giudice et al., 2011; Gitti et al., 2011; Hong et al., 2011; Hsiao et al., 2011; Jeong et al., 2011; Lan et al., 2011; Chen et al., 2012; Lucke et al., 2012; Matveychuk et al., 2012; Braun et al., 2013; Cortés-Torres et al., 2013; Hombach et al., 2013; Mirsaeidi et al., 2013; Saifi et al., 2013; Chou et al., 2014; Lin et al., 2014; Sookan and Coovadia, 2014; Wu et al., 2014; Bainomugisa et al., 2015; Chiang et al., 2015; Kim et al., 2015; Lee et al., 2015; Manika et al., 2015; Ng et al., 2015; Shao et al., 2015; Sheu et al., 2015; Blanc et al., 2016; Kodana et al., 2016; Nasr Esfahani et al., 2016; Ose et al., 2016; Riello et al., 2016; Agizew et al., 2017; Brown-Elliott and Wallace, 2017, 2021; Desikan et al., 2017; Pang et al., 2017; Park et al., 2017; Adzic-Vukicevic et al., 2018; Loizos et al., 2018; Luo et al., 2018; Naito et al., 2018; Nasiri et al., 2018; Tan et al., 2018; Davari et al., 2019; Fang et al., 2019; Gomathy et al., 2019; Marques et al., 2019; Modrá et al., 2019; Mortazavi et al., 2019; Pedrero et al., 2019; Xu et al., 2019; Feysia et al., 2020; Hara et al., 2020; Huang et al., 2020; López-Roa et al., 2020; Takenaka et al., 2020; Thomson et al., 2020; Abate et al., 2021; Ahn et al., 2021; Donohue, 2021; Huang et al., 2021; Kim M. J. et al., 2021; Kim Y. G. et al., 2021; Liu et al., 2021; Mahdavi et al., 2021; Ose et al., 2021; Thangavelu et al., 2021; Urabe et al., 2021; Chai et al., 2022; Das et al., 2022; Gaballah et al., 2022; Gao et al., 2022; He et al., 2022; Lee et al., 2022; Lin et al., 2022), while 16 published studies were used to analyze the prevalence of *M. kansasi* in environmental isolates (McSwiggan and Collins, 1974; Engel et al., 1980; Gruft et al., 1981; Wright et al., 1985; Kubalek and Mysak, 1996; Slosárek et al., 1996; Iivanainen et al., 1999; Santos et al., 2005; Ghaemi et al., 2006; Thomas et al., 2006; Sharma et al., 2007; Parashar et al., 2009; Adrados et al., 2011; Kwenda et al., 2015; Moghaddam et al., 2022).

Figure 1 illustrates the review and article selection process based on the Preferred Reporting Items for Systematic Review and Meta-Analyses (PRISMA) statement. Supplementary Table S1 provides the characteristics of the included studies and the quality control analysis score, while Supplementary Table S2 presents the details of the answers to the JBI checklist questions for quality control.

### Meta-analysis

**Figure 2:**
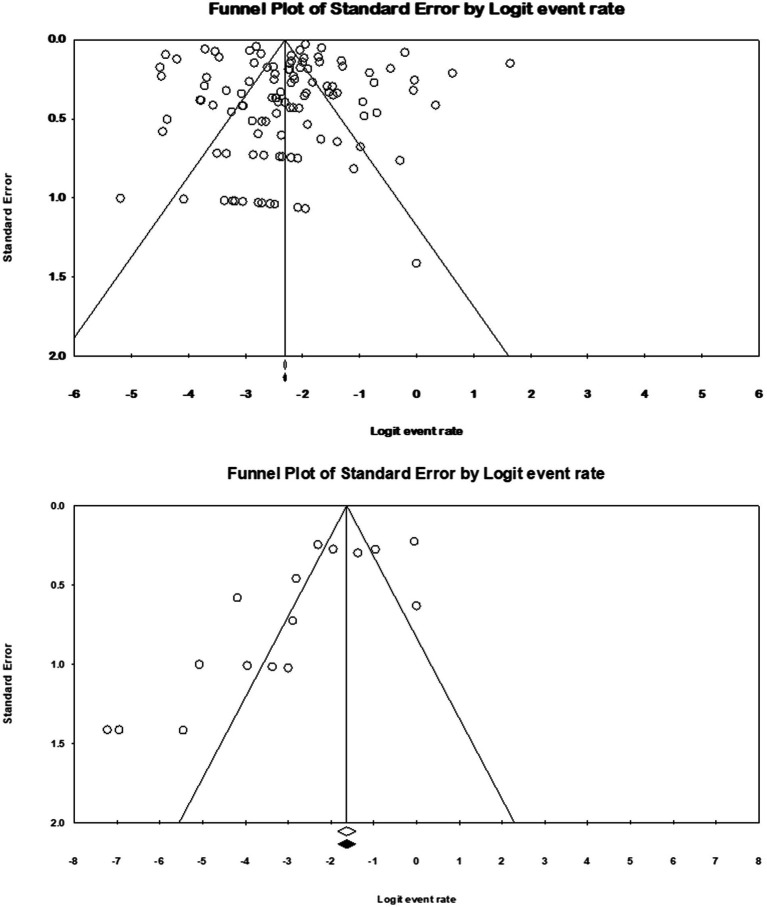
Funnel plots for identification of publication bias in clinical and environmental isolates.

Funnel plots (Figure 2) showed publication bias for the prevalence result of *M. kansasi* in NTM clinical isolates and environmental isolates. Begg’s test was also used to indicate publication bias for the prevalence results (*p* = 0.11 for prevalence in NTM isolates and *p* = 0.079 for prevalence in environmental isolates).

Sensitivity analysis was conducted, and the results demonstrated that none of the studies influenced the prevalence of *M. kansasi* in NTM clinical isolates and environmental isolates.

This study included 111 articles that reported the prevalence of *M. kansasi* in NTM isolates. The prevalence of *M. kansasi* in these isolates was 9.4% (95% CI: 0.07–0.11%; I^2^ = 97.75%; *p* < 0.001).

**Figure 3:**
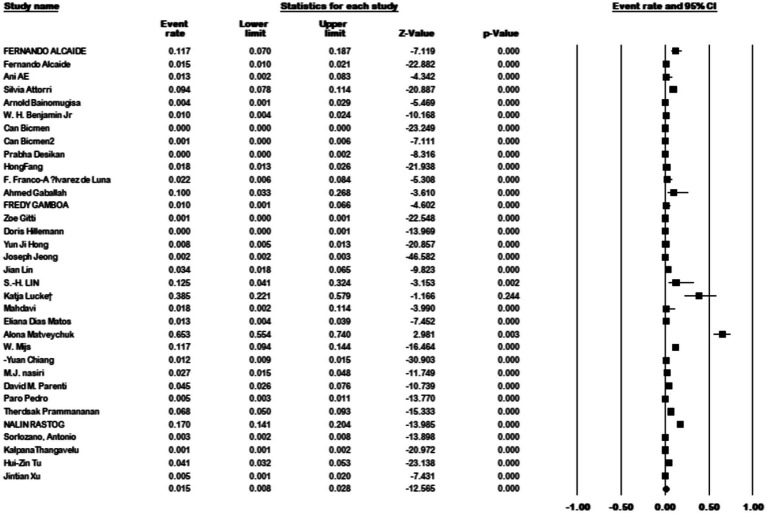
Forest plot showing the prevalence of *M. kansasii* in total clinical isolates.

**Figure 4:**
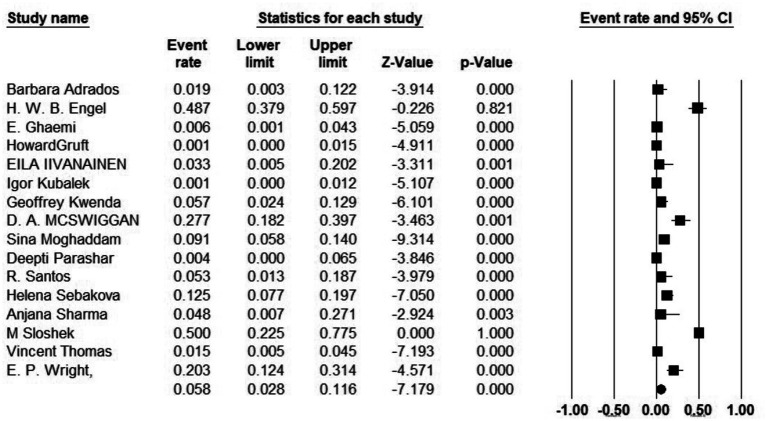
Forest plot showing the prevalence of *M. kansasii* in environmental isolates.

**Figure 5:**
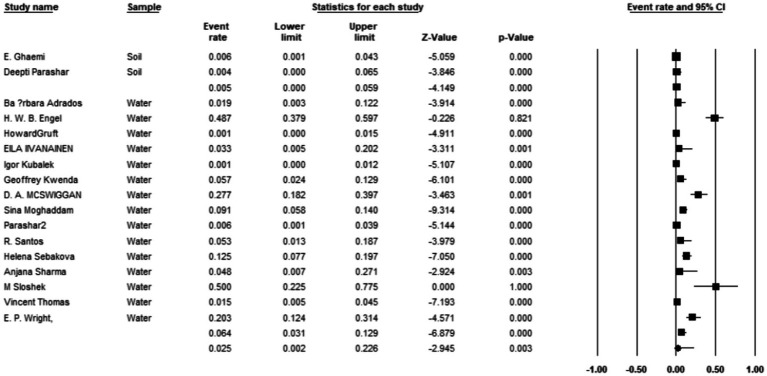
Forest plot showing the prevalence of *M. kansasii* in different environmental isolates.

Additionally, 34 articles provided information on the total number of collected isolates, and the prevalence of *M. kansasi* was found to be 1.5% (95% CI: 0.08–0.028%; I^2^ = 98.44%; *p* < 0.001) (Figure 3). Sixteen articles investigated the prevalence of *M. kansasi* in environmental isolates, including water and soil. The prevalence of *M. kansasi* in these isolates was 5.8% (95% CI: 0.028–0.116%; I^2^ = 90.518%; *p* < 0.001) (Figure 4). Two articles examined the prevalence of *M. kansasi* in soil, resulting in a prevalence rate of 0.5% (95% CI: 0.000–0.059). Fifteen studies reported the prevalence of *M. kansasi* in water, which was found to be 6.4% (95% CI: 0.031–0.129; *p* < 0.001) (Figure 5).

### Subgroup analysis of prevalence of *Mycobacterium kansasii* in NTM isolates

**Figure 6:**
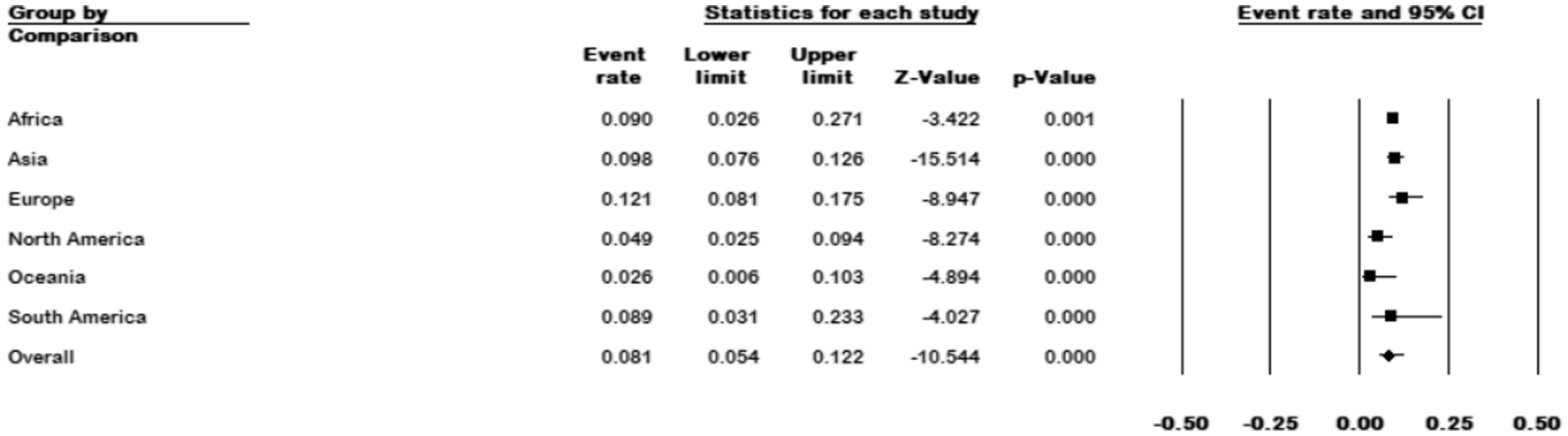
Forest plot showing the prevalence of *M. kansasii* in NTM isolates for different continents.

Among the 111 studies that reported the prevalence of *M. kansasi* in NTM isolates, 64 were conducted in Asia, 25 in Europe, 10 in North America, 6 in Africa, 4 in South America, and 2 in Oceania. The prevalence of *M. kansasi* was highest in Europe with 12.1% (95% CI 0.08–0.17) and lowest in North America with 2.6% (95% CI 0.006–0.0103) (*p* < 0.001) (Figure 6).

**Figure 7:**
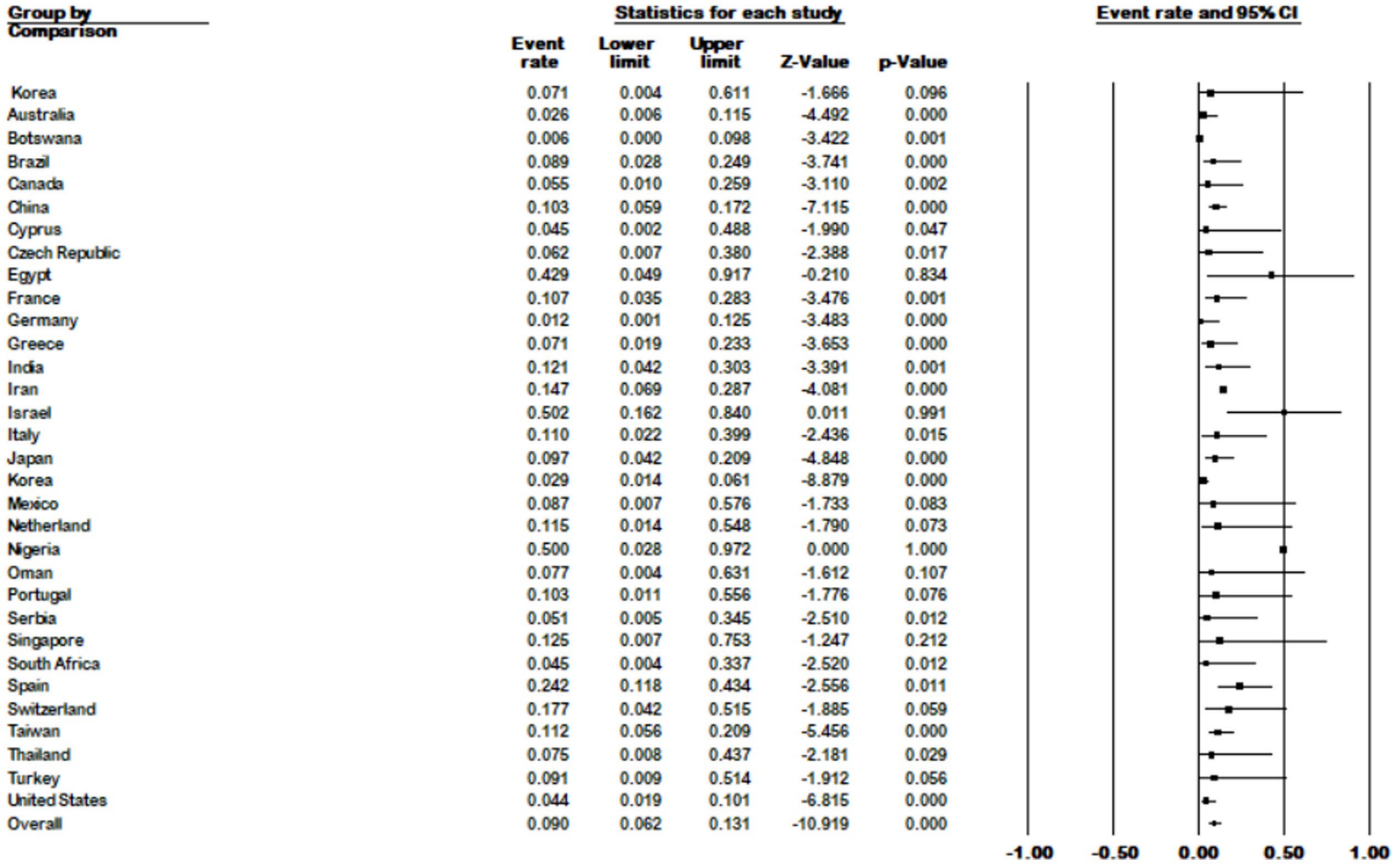
Forest plot showing the prevalence of *M. kansasii* in NTM isolates for different countries.

The results of the country subgroup meta-analysis showed that Israel had the highest prevalence of *M. kansasi* in NTM with 50% (95% CI 0.162–0.84), while Botswana had the lowest with 0.6% (95% CI 0.00–0.098) (*p* < 0.001) (Figure 7).

**Figure 8:**
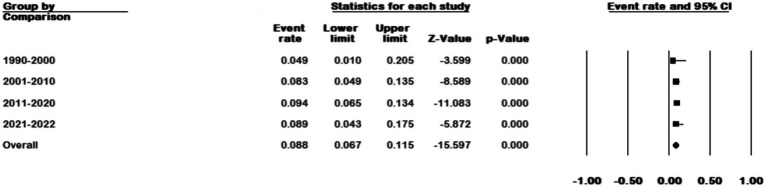
Forest plot showing the prevalence of *M. kansasii* in NTM isolates in four periods.

We divided the sample collection time into four periods and analyzed studies whose sample collection time matched our grouping. The results showed an increase in prevalence from 4.9% (95% CI 0.01–0.20) in 1990–2000 to 8.9% (95% CI 0.043–0.175) in 2021–2022 (*p* < 0.001) (Figure 8).

**Figure 9:**
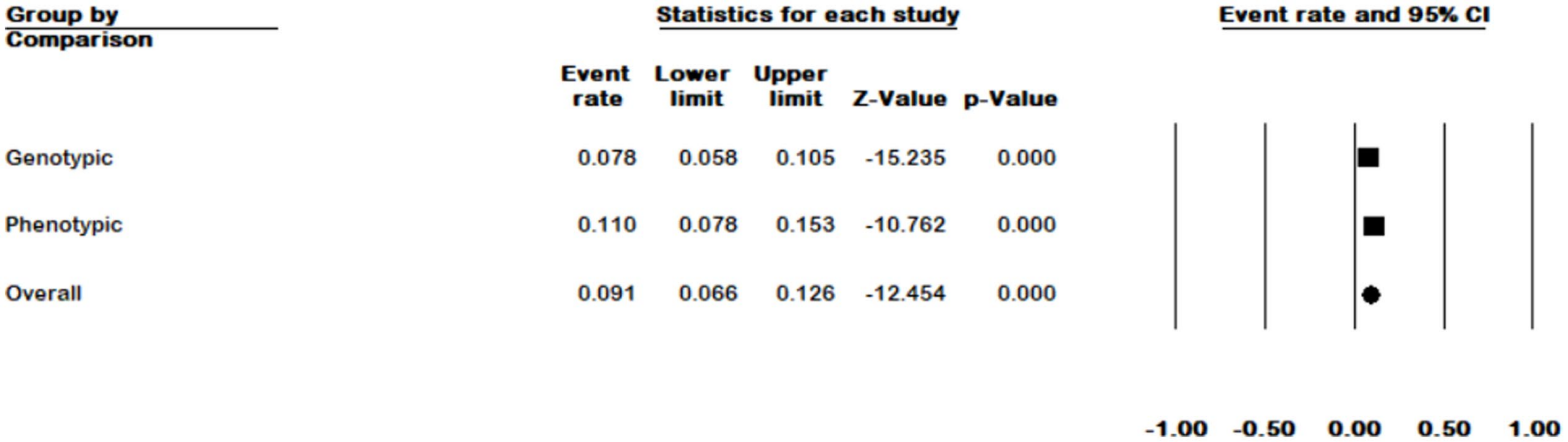
Forest plot showing the prevalence of *M. kansasii* in NTM isolates for two detection methods.

In general, we divided *M. kansasi* identification methods into two categories: phenotypic methods including culture characteristics, biochemical methods, MALDI-TOF, and HPLC; and genotypic methods such as sequencing, hybridization, and using probes. According to genotypic methods, the prevalence of *M. kansasi* in NTM isolates was 7.8% (95% CI 0.058–0.105), and according to phenotypic methods, it was 11% (95% CI 0.078–0.153) (*p* = 753) (Figure 9).

### Subgroup analysis of prevalence of *Mycobacterium kansasii* in water

**Figure 10:**
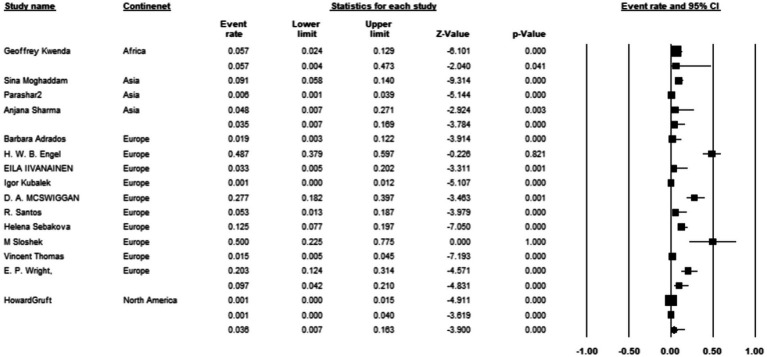
Forest plot showing the prevalence of *M. kansasii* in water sample in different continents.

Among the 15 studies reporting the prevalence of *M. kansasi* in water, 3 were conducted in Asia, 10 in Europe, 1 in North America, and 1 in Africa. Europe exhibited the highest prevalence of *M. kansasi* at 9.7% (95% CI 0.042–0.21, *p* < 0.001) (Figure 10).

**Figure 11:**
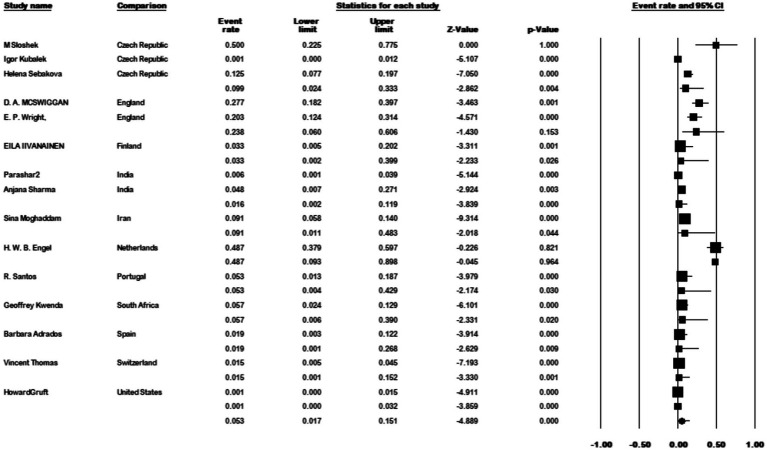
Forest plot showing the prevalence of *M. kansasii* in water sample in different countries.

**Figure 12:**
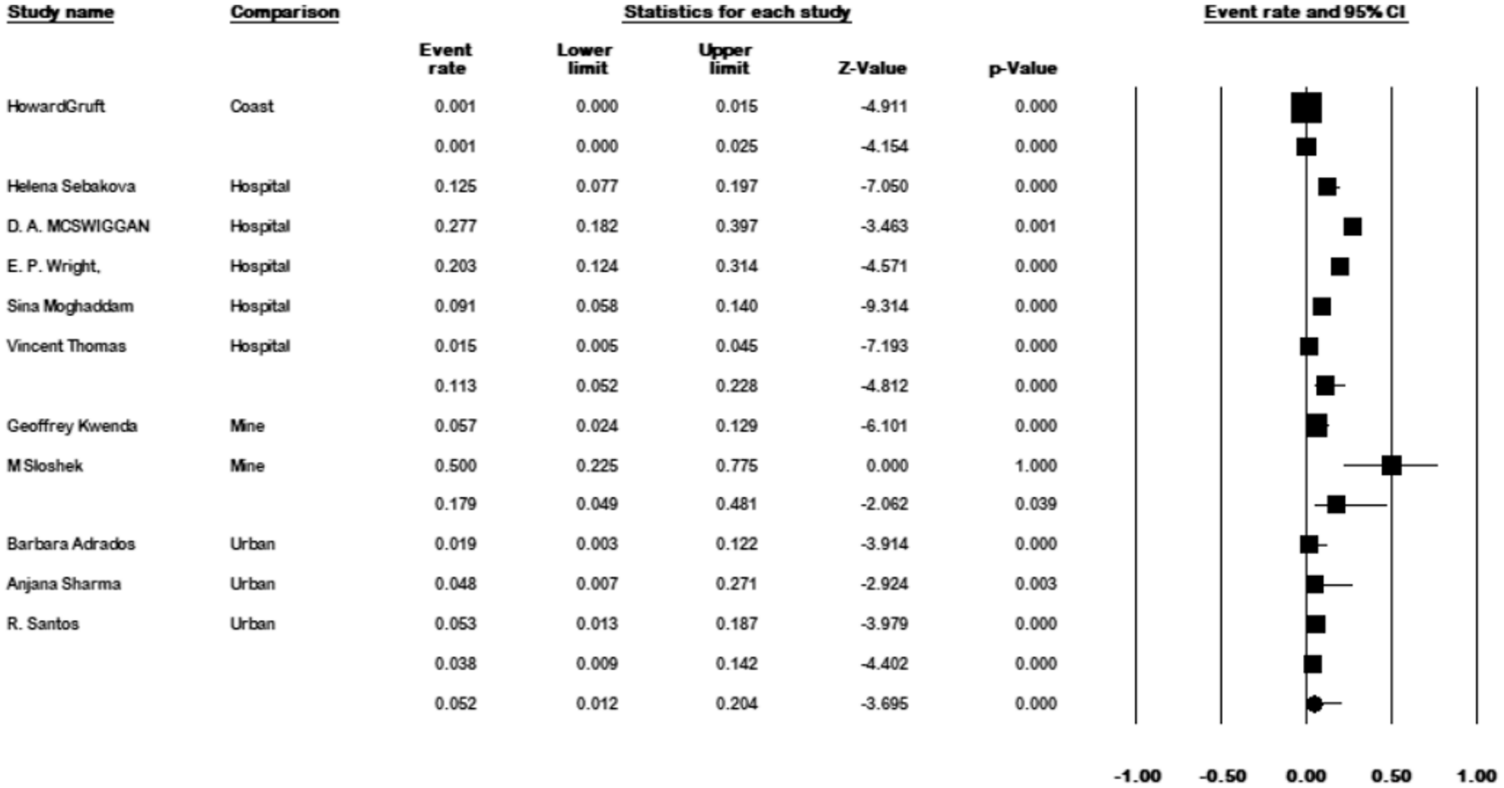
Forest plot showing the prevalence of *M. kansasii* in water sample in different locations.

The results of the country subgroup meta-analysis demonstrated that the Netherlands had the highest prevalence of *M. kansasi* in water, with a rate of 48.7% (95% CI 0.093–0.898, *p* < 0.001) (Figure 11). Furthermore, a subgroup analysis was conducted based on the location of water sample collection. The results indicated a particularly high prevalence of 17.9% (95% CI 0.048–0.484) in mine locations, compared to other sampling sites (*p* < 0.001) (Figure 12).

## Discussion

*M. kansasii* was among the first non-tuberculous mycobacteria (NTM) to be identified as a respiratory pathogen in humans (Akram and Rawla, 2023). It is the sixth most commonly encountered NTM globally, although there is limited information on its prevalence in various sources and countries (Bakuła et al., 2013). This systematic review and meta-analysis sought to investigate the prevalence of *M. kansasii*. Our findings revealed a global prevalence of 9.4% among NTM isolates.

Studies have documented distinct regional differences in the prevalence of *M. kansasii* pulmonary disease, ranging from 3 to 70% worldwide (Guo et al., 2022). Notably, certain regions exhibit a higher prevalence of this bacterium. Our data analysis highlighted that Europe had the highest prevalence at 12.1%, followed by Asia at 9.8%, while Oceania had the lowest prevalence at 2.6% among the continents examined. The reason for the higher prevalence of this bacterium in Europe may be due to the lack of advanced diagnostic facilities in developing countries along with a larger susceptible population due to the aging population in Europe.

Studies have revealed distinct regional differences in the prevalence of *M. kansasii* pulmonary disease, with a relatively high incidence observed in Brazil, Australia, Poland, and the United Kingdom (Zhang et al., 2023). The meta-analysis conducted by Okoi et al. identified *M. kansasii* as the most common cause of pulmonary NTM disease in a sub-Saharan African country (Okoi et al., 2017), with a prevalence rate of 69.2%. Additionally, Khosravi et al. reported frequencies ranging from 13 to 17% for this pathogen among all NTM isolates in Iran (Khosravi et al., 2020). Similarly, Morimoto et al. reported *M. kansasii* as the most prevalent form of NTM in Japan, with a prevalence rate of 43.6% (Morimoto et al., 2017).

Our analysis yielded the highest prevalence rates in Israel, Nigeria, and Egypt, with percentages of 50.2, 50, and 42.9%, respectively. On the other hand, Botswana and Germany exhibited the lowest prevalence rates at 0.06 and 1.2%, respectively.

Overall, NTM diseases are increasingly prevalent worldwide, potentially due to a rising population susceptible to weakened immune systems, organ transplantation, aging, changes in the environment favoring NTM development, and reduced anti-mycobacterial immunity following failed tuberculosis treatment (Chin et al., 2020). To date, there is no comprehensive global study investigating the temporal prevalence of *M. kansasii*. However, existing studies that have examined its prevalence over time have reported a significant increase in the prevalence of this bacterium. We divided the time of sample collection into four periods (1990–2000, 2001–2010, 2011–2020, and 2021–2022). The results of data analysis showed prevalence rates of 49, 83, 94, and 89%, respectively. Overall, our results demonstrated a significant increase in the prevalence of *M. kansasii* over time.

In this study, we analyzed the prevalence of *M. kansasii* in water samples. The prevalence of *M. kansasii* in water samples was found to be 5.8%, while the prevalence in all clinical isolates was 1.5%. This indicates a high prevalence of this microorganism in water samples, suggesting that water serves as a reservoir for this bacterium. Furthermore, the ability of *M. kansasii* to form biofilms may result in the release of this microorganism into water, posing a risk to consumers through drinking or inhalation of aerosols from showers, swimming pools, spas, and other water systems (Muñoz-Egea et al., 2023). Therefore, monitoring water samples is crucial for infection control.

Mining has long been associated with diseases caused by NTM, implying that exposure to mining dust may contribute to NTM transmission (Marras and Daley, 2002). As a result, miners may face a higher risk of exposure to potentially pathogenic environmental mycobacteria compared to workers in other occupations. In our study, water samples isolated from the mine had the highest prevalence rate at 17.9%, underscoring the significance of this location as a potential risk factor for *M. kansasii* transmission.

Although we made efforts to conduct a comprehensive search, it is possible that not all of the relevant existing literature was included. One potential limitation of this meta-analysis is that gray literature was not included in the search strategy. As a result, relevant studies or data that may have been available through gray literature channels, such as conference proceedings or unpublished dissertations, might have been inadvertently overlooked. This limitation could potentially introduce selection bias and limit the comprehensiveness of the findings. The relatively high heterogeneity between studies was another limitation of the present study. To address this, we conducted subgroup analysis to explore the sources of heterogeneity and minimize its impact on the results.

## Conclusion

*Mycobacterium kansasii* is a prevalent causative agent of nontuberculous mycobacterial lung disease globally. Our results have highlighted a substantial prevalence of *M. kansasii* in clinical isolates, emphasizing the urgent need for heightened attention from health authorities, physicians, and microbiologists.

Furthermore, our investigation into the prevalence over time has revealed a significant increase in the occurrence of this bacterium, underscoring the importance of enhanced identification and control measures within infection control strategies to mitigate its further spread. Additionally, our study has demonstrated a higher prevalence of *M. kansasii* in water samples, further accentuating the significance of screening these samples for this microorganism as a preventive measure against the associated disease.

## Data availability statement

The original contributions presented in the study are included in the article/Supplementary material, further inquiries can be directed to the corresponding author.

## Author contributions

NN: Conceptualization, Formal analysis, Methodology, Software, Writing – original draft, Writing – review & editing. NB: Data curation, Writing – review & editing. FG: Data curation, Writing – review & editing. SR: Investigation, Methodology, Writing – review & editing. FJ: Supervision, Writing – review & editing.
