# Supplementary material for: Prevalence of Mycobacterium kansasii in clinical and environmental isolates, a systematic review and meta-analysis
Source: Front Microbiol. 2024 Feb 19;15:1321273. doi: 10.3389/fmicb.2024.1321273 (PMC10911025; doi:10.3389/fmicb.2024.1321273)
Supplement: Supplementary file 2 [file Table_2.DOCX]

| No of Study | Q1 | Q2 | Q3 | Q4 | Q5 | Q6 | Q7 | Q8 | Q9 | Total |
| --- | --- | --- | --- | --- | --- | --- | --- | --- | --- | --- |
| 1 | **N** | **Y** | **N** | **Y** | **Y** | **N** | **Y** | **N** | **Y** | **5** |
| 2 | **N** | **Y** | **N** | **Y** | **Y** | **N** | **Y** | **Y** | **Y** | **6** |
| 3 | **N** | **N** | **N** | **Y** | **Y** | **Y** | **Y** | **Y** | **Y** | **6** |
| 4 | **Y** | **Y** | **Y** | **Y** | **Y** | **Y** | **Y** | **Y** | **Y** | **9** |
| 5 | **Y** | **N** | **Y** | **Y** | **Y** | **N** | **Y** | **N** | **Y** | **6** |
| 6 | **Y** | **Y** | **Y** | **Y** | **Y** | **N** | **N** | **Y** | **Y** | **7** |
| 7 | **Y** | **Y** | **Y** | **Y** | **Y** | **N** | **Y** | **Y** | **Y** | **8** |
| 8 | **Y** | **Y** | **Y** | **Y** | **Y** | **Y** | **Y** | **N** | **Y** | **8** |
| 9 | **Y** | **Y** | **Y** | **Y** | **Y** | **N** | **Y** | **Y** | **Y** | **8** |
| 10 | **Y** | **Y** | **Y** | **Y** | **Y** | **N** | **Y** | **Y** | **Y** | **8** |
| 11 | **N** | **Y** | **N** | **Y** | **Y** | **N** | **Y** | **N** | **Y** | **5** |
| 12 | **Y** | **Y** | **Y** | **Y** | **Y** | **N** | **Y** | **N** | **Y** | **7** |
| 13 | **Y** | **Y** | **Y** | **Y** | **Y** | **N** | **Y** | **N** | **Y** | **7** |
| 14 | **Y** | **Y** | **Y** | **N** | **N** | **N** | **Y** | **Y** | **Y** | **6** |
| 15 | **Y** | **Y** | **Y** | **Y** | **Y** | **Y** | **Y** | **N** | **Y** | **8** |
| 16 | **Y** | **Y** | **Y** | **Y** | **Y** | **N** | **Y** | **N** | **Y** | **7** |
| 17 | **Y** | **Y** | **Y** | **Y** | **Y** | **Y** | **Y** | **Y** | **Y** | **9** |
| 18 | **Y** | **Y** | **Y** | **Y** | **Y** | **Y** | **Y** | **Y** | **Y** | **9** |
| 19 | **Y** | **Y** | **Y** | **Y** | **Y** | **Y** | **Y** | **N** | **Y** | **8** |
| 20 | **Y** | **N** | **Y** | **Y** | **N** | **N** | **N** | **N** | **Y** | **4** |
| 21 | **Y** | **N** | **Y** | **N** | **N** | **N** | **N** | **N** | **Y** | **3** |
| 22 | **Y** | **Y** | **Y** | **N** | **N** | **Y** | **Y** | **Y** | **Y** | **7** |
| 23 | **Y** | **Y** | **Y** | **Y** | **N** | **Y** | **Y** | **N** | **Y** | **7** |
| 24 | **Y** | **Y** | **Y** | **N** | **N** | **N** | **Y** | **Y** | **Y** | **6** |
| 25 | **Y** | **Y** | **Y** | **N** | **N** | **N** | **Y** | **Y** | **Y** | **6** |
| 26 | **N** | **Y** | **N** | **Y** | **N** | **Y** | **Y** | **Y** | **Y** | **5** |
| 27 | **Y** | **Y** | **Y** | **N** | **N** | **Y** | **Y** | **Y** | **Y** | **7** |
| 28 | **Y** | **Y** | **Y** | **Y** | **Y** | **Y** | **Y** | **N** | **Y** | **8** |
| 29 | **Y** | **Y** | **Y** | **Y** | **Y** | **Y** | **Y** | **N** | **Y** | **8** |
| 30 | **Y** | **Y** | **Y** | **Y** | **Y** | **N** | **Y** | **N** | **Y** | **7** |
| 31 | **N** | **Y** | **N** | **Y** | **Y** | **Y** | **Y** | **Y** | **Y** | **7** |
| 32 | **Y** | **Y** | **Y** | **Y** | **Y** | **Y** | **Y** | **N** | **Y** | **8** |
| 33 | **N** | **N** | **N** | **N** | **N** | **N** | **N** | **Y** | **Y** | **2** |
| 33 | **Y** | **Y** | **Y** | **Y** | **Y** | **N** | **Y** | **Y** | **Y** | **8** |
| 34 | **Y** | **Y** | **Y** | **Y** | **Y** | **N** | **Y** | **Y** | **Y** | **8** |
| 35 | **N** | **Y** | **N** | **Y** | **Y** | **Y** | **Y** | **Y** | **Y** | **7** |
| 36 | **Y** | **Y** | **Y** | **Y** | **Y** | **N** | **Y** | **N** | **Y** | **7** |
| 37 | **N** | **Y** | **N** | **Y** | **Y** | **N** | **Y** | **N** | **Y** | **5** |
| 38 | **N** | **Y** | **N** | **Y** | **Y** | **N** | **Y** | **N** | **Y** | **5** |
| 39 | **Y** | **Y** | **Y** | **Y** | **Y** | **N** | **Y** | **Y** | **Y** | **8** |
| 40 | **Y** | **Y** | **Y** | **Y** | **Y** | **Y** | **Y** | **Y** | **Y** | **9** |
| 41 | **Y** | **Y** | **Y** | **Y** | **Y** | **Y** | **Y** | **Y** | **Y** | **9** |
| 42 | **Y** | **Y** | **Y** | **N** | **N** | **Y** | **Y** | **N** | **Y** | **6** |
| 43 | **Y** | **Y** | **Y** | **Y** | **Y** | **Y** | **Y** | **N** | **Y** | **8** |
| 44 | **Y** | **Y** | **Y** | **Y** | **Y** | **N** | **Y** | **N** | **Y** | **7** |
| 45 | **Y** | **N** | **Y** | **N** | **N** | **N** | **N** | **N** | **Y** | **3** |
| 46 | **Y** | **Y** | **Y** | **N** | **N** | **Y** | **Y** | **N** | **Y** | **6** |
| 47 | **Y** | **Y** | **Y** | **Y** | **Y** | **N** | **Y** | **N** | **Y** | **7** |
| 48 | **Y** | **N** | **Y** | **Y** | **Y** | **N** | **N** | **Y** | **Y** | **6** |
| 49 | **Y** | **Y** | **Y** | **Y** | **Y** | **Y** | **Y** | **Y** | **Y** | **9** |
| 50 | **Y** | **Y** | **Y** | **Y** | **Y** | **N** | **Y** | **N** | **Y** | **7** |
| 51 | **Y** | **Y** | **Y** | **Y** | **N** | **N** | **Y** | **N** | **N** | **5** |
| 52 | **Y** | **Y** | **Y** | **N** | **N** | **N** | **Y** | **N** | **Y** | **5** |
| 53 | **Y** | **Y** | **Y** | **Y** | **Y** | **N** | **Y** | **Y** | **Y** | **8** |
| 54 | **Y** | **Y** | **Y** | **Y** | **N** | **N** | **Y** | **N** | **N** | **5** |
| 55 | **Y** | **Y** | **Y** | **N** | **N** | **N** | **Y** | **Y** | **Y** | **6** |
| 56 | **Y** | **Y** | **Y** | **Y** | **Y** | **N** | **Y** | **N** | **Y** | **7** |
| 57 | **N** | **Y** | **N** | **Y** | **N** | **N** | **Y** | **Y** | **N** | **4** |
| 58 | **N** | **Y** | **N** | **Y** | **Y** | **Y** | **Y** | **Y** | **Y** | **7** |
| 59 | **Y** | **Y** | **Y** | **Y** | **Y** | **Y** | **Y** | **Y** | **Y** | **9** |
| 60 | **Y** | **Y** | **Y** | **N** | **N** | **N** | **Y** | **Y** | **Y** | **6** |
| 61 | **N** | **Y** | **N** | **Y** | **Y** | **N** | **Y** | **Y** | **N** | **5** |
| 62 | **Y** | **Y** | **Y** | **Y** | **Y** | **N** | **Y** | **N** | **Y** | **7** |
| 63 | **Y** | **Y** | **Y** | **Y** | **Y** | **N** | **Y** | **Y** | **Y** | **8** |
| 64 | **Y** | **Y** | **Y** | **Y** | **Y** | **N** | **Y** | **N** | **Y** | **7** |
| 65 | **Y** | **Y** | **Y** | **Y** | **Y** | **N** | **Y** | **N** | **Y** | **7** |
| 66 | **Y** | **Y** | **Y** | **Y** | **Y** | **N** | **Y** | **N** | **Y** | **7** |
| 67 | **Y** | **N** | **Y** | **N** | **N** | **N** | **N** | **Y** | **Y** | **4** |
| 68 | **Y** | **Y** | **Y** | **Y** | **Y** | **N** | **Y** | **N** | **Y** | **7** |
| 69 | **Y** | **Y** | **Y** | **N** | **N** | **N** | **Y** | **N** | **Y** | **5** |
| 70 | **Y** | **N** | **Y** | **N** | **N** | **Y** | **N** | **N** | **N** | **3** |
| 71 | **Y** | **Y** | **Y** | **Y** | **Y** | **Y** | **Y** | **N** | **Y** | **8** |
| 72 | **Y** | **Y** | **Y** | **N** | **N** | **N** | **Y** | **Y** | **Y** | **6** |
| 73 | **Y** | **Y** | **Y** | **Y** | **Y** | **Y** | **Y** | **Y** | **Y** | **9** |
| 74 | **Y** | **Y** | **Y** | **Y** | **N** | **Y** | **Y** | **Y** | **Y** | **8** |
| 75 | **Y** | **Y** | **Y** | **N** | **N** | **N** | **Y** | **Y** | **Y** | **7** |
| 76 | **Y** | **Y** | **Y** | **Y** | **N** | **Y** | **Y** | **Y** | **N** | **7** |
| 77 | **Y** | **N** | **Y** | **N** | **N** | **Y** | **N** | **Y** | **Y** | **5** |
| 78 | **N** | **Y** | **N** | **Y** | **Y** | **N** | **Y** | **Y** | **Y** | **7** |
| 79 | **Y** | **N** | **Y** | **N** | **N** | **N** | **N** | **N** | **Y** | **3** |
| 80 | **Y** | **Y** | **Y** | **Y** | **Y** | **Y** | **Y** | **N** | **Y** | **8** |
| 81 | **Y** | **Y** | **Y** | **Y** | **Y** | **Y** | **Y** | **Y** | **N** | **8** |
| 82 | **Y** | **Y** | **Y** | **N** | **N** | **Y** | **Y** | **Y** | **Y** | **7** |
| 83 | **Y** | **Y** | **Y** | **N** | **N** | **N** | **Y** | **Y** | **Y** | **6** |
| 84 | **Y** | **Y** | **Y** | **Y** | **Y** | **Y** | **Y** | **Y** | **Y** | **9** |
| 85 | **Y** | **Y** | **Y** | **Y** | **Y** | **N** | **Y** | **N** | **Y** | **7** |
| 86 | **Y** | **Y** | **Y** | **Y** | **N** | **Y** | **Y** | **N** | **Y** | **7** |
| 87 | **Y** | **Y** | **Y** | **N** | **N** | **Y** | **Y** | **Y** | **Y** | **7** |
| 88 | **Y** | **N** | **Y** | **N** | **N** | **N** | **N** | **Y** | **Y** | **4** |
| 89 | **N** | **Y** | **N** | **Y** | **Y** | **N** | **Y** | **N** | **Y** | **5** |
| 90 | **Y** | **Y** | **Y** | **Y** | **N** | **Y** | **Y** | **Y** | **Y** | **8** |
| 91 | **Y** | **Y** | **Y** | **Y** | **Y** | **Y** | **Y** | **Y** | **Y** | **9** |
| 92 | **Y** | **Y** | **Y** | **Y** | **Y** | **Y** | **Y** | **N** | **Y** | **8** |
| 93 | **N** | **Y** | **N** | **Y** | **Y** | **Y** | **Y** | **Y** | **Y** | **7** |
| 94 | **Y** | **Y** | **Y** | **N** | **N** | **N** | **Y** | **N** | **Y** | **5** |
| 95 | **Y** | **Y** | **Y** | **N** | **N** | **N** | **Y** | **N** | **Y** | **5** |
| 96 | **Y** | **Y** | **Y** | **Y** | **Y** | **N** | **Y** | **N** | **Y** | **7** |
| 97 | **Y** | **Y** | **Y** | **Y** | **Y** | **N** | **Y** | **N** | **Y** | **7** |
| 98 | **Y** | **Y** | **Y** | **Y** | **Y** | **N** | **Y** | **N** | **Y** | **7** |
| 99 | **Y** | **Y** | **Y** | **Y** | **Y** | **Y** | **Y** | **N** | **Y** | **8** |
| 100 | **Y** | **Y** | **Y** | **Y** | **Y** | **N** | **Y** | **N** | **Y** | **7** |
| 101 | **Y** | **Y** | **Y** | **Y** | **Y** | **N** | **Y** | **Y** | **Y** | **7** |
| 102 | **Y** | **Y** | **Y** | **N** | **N** | **N** | **Y** | **Y** | **Y** | **6** |
| 103 | **Y** | **Y** | **Y** | **Y** | **Y** | **N** | **Y** | **N** | **Y** | **7** |
| 104 | **Y** | **Y** | **Y** | **Y** | **N** | **N** | **Y** | **N** | **Y** | **6** |
| 105 | **Y** | **Y** | **Y** | **N** | **N** | **Y** | **Y** | **N** | **Y** | **6** |
| 106 | **Y** | **Y** | **Y** | **Y** | **Y** | **N** | **Y** | **Y** | **Y** | **8** |
| 107 | **Y** | **Y** | **Y** | **N** | **N** | **N** | **Y** | **N** | **Y** | **5** |
| 108 | **Y** | **Y** | **Y** | **N** | **N** | **N** | **Y** | **Y** | **Y** | **6** |
| 109 | **N** | **Y** | **N** | **Y** | **Y** | **Y** | **Y** | **N** | **Y** | **6** |
| 110 | **Y** | **Y** | **Y** | **Y** | **N** | **Y** | **Y** | **Y** | **Y** | **8** |
| 111 | **Y** | **Y** | **Y** | **Y** | **Y** | **N** | **Y** | **Y** | **Y** | **8** |
| 112 | **Y** | **Y** | **N** | **N** | **Y** | **Y** | **Y** | **Y** | **Y** | **7** |
| 113 | **Y** | **Y** | **Y** | **Y** | **Y** | **Y** | **Y** | **N** | **Y** | **8** |
| 114 | **Y** | **Y** | **Y** | **Y** | **Y** | **N** | **Y** | **N** | **Y** | **7** |
| 115 | **Y** | **Y** | **Y** | **Y** | **Y** | **Y** | **Y** | **Y** | **Y** | **9** |
| 116 | **Y** | **N** | **Y** | **N** | **N** | **N** | **N** | **N** | **Y** | **3** |
| 117 | **Y** | **Y** | **Y** | **Y** | **Y** | **N** | **Y** | **Y** | **Y** | **8** |
| 118 | **Y** | **Y** | **Y** | **Y** | **Y** | **N** | **Y** | **Y** | **N** | **7** |
| Environment |  |  |  |  |  |  |  |  |  |  |
| 1 | **Y** | **N** | **Y** | **N** | **N** | **N** | **N** | **N** | **Y** | **3** |
| 2 | **Y** | **Y** | **Y** | **N** | **N** | **Y** | **Y** | **N** | **Y** | **6** |
| 3 | **N** | **Y** | **N** | **Y** | **Y** | **N** | **Y** | **Y** | **Y** | **6** |
| 4 | **N** | **N** | **N** | **Y** | **Y** | **N** | **N** | **N** | **Y** | **3** |
| 5 | **Y** | **Y** | **Y** | **Y** | **Y** | **Y** | **Y** | **N** | **Y** | **8** |
| 6 | **Y** | **Y** | **Y** | **Y** | **N** | **N** | **Y** | **Y** | **Y** | **7** |
| 7 | **Y** | **Y** | **Y** | **Y** | **N** | **Y** | **Y** | **N** | **Y** | **7** |
| 8 | **N** | **Y** | **N** | **Y** | **Y** | **Y** | **Y** | **N** | **Y** | **6** |
| 9 | **Y** | **Y** | **Y** | **Y** | **Y** | **Y** | **Y** | **N** | **Y** | **8** |
| 10 | **Y** | **Y** | **Y** | **Y** | **Y** | **Y** | **Y** | **Y** | **Y** | **9** |
| 11 | **Y** | **Y** | **Y** | **Y** | **N** | **Y** | **Y** | **N** | **Y** | **7** |
| 12 | **Y** | **Y** | **Y** | **Y** | **Y** | **Y** | **Y** | **Y** | **Y** | **9** |
| 13 | **N** | **Y** | **Y** | **Y** | **Y** | **Y** | **Y** | **N** | **Y** | **7** |
| 14 | **N** | **Y** | **Y** | **Y** | **N** | **N** | **Y** | **N** | **Y** | **5** |
| 15 | **N** | **U** | **N** | **Y** | **Y** | **Y** | **U** | **U** | **Y** | **4** |
| 16 | **N** | **Y** | **N** | **Y** | **Y** | **Y** | **Y** | **U** | **Y** | **6** |
